# The Treatment of Tapeworm

**Published:** 1877-11

**Authors:** 


The Treatment of Tapeworm.—Prof. Mosler has been advocating a system of treating tapeworm, which, according to a Swiss medical journal, has been attended with remarkable success. Its chief characteristic is the injection of large quantities of warm water into the colon, after the administration of the anthelmintic. The diet is first regulated, food being given which is supposed to be distasteful to the tapeworm—bilberry tea, herrings, sour cucumber, salted meats. The intestine having been, as far as possible, emptied by laxatives, a dose of the extract of pomegranate bark is administered, prepared from the fresh bark, and then a large quantity of warm water is injected into the rectum. The theory is that the worm, previously brought down into the colon, is prevented by the water from attaching itself to the wall, and is brought away by the liquid on its escape. It is asserted that in every case in which this treatment was adopted, the head of the worm was removed.

